# High‐efficient generation of VCAM‐1^+^ mesenchymal stem cells with multidimensional superiorities in signatures and efficacy on aplastic anaemia mice

**DOI:** 10.1111/cpr.12862

**Published:** 2020-06-29

**Authors:** Yimeng Wei, Leisheng Zhang, Ying Chi, Xiang Ren, Yuchen Gao, Baoquan Song, Chengwen Li, Zhibo Han, Lei Zhang, Zhongchao Han

**Affiliations:** ^1^ State Key Laboratory of Experimental Hematology & National Clinical Research Center for Blood Disease Institute of Hematology & Blood Diseases Hospital Chinese Academy of Medical Sciences & Peking Union Medical College Tianjin China; ^2^ The Postdoctoral Research Station School of Medicine Nankai University Tianjin China; ^3^ The Enterprise Postdoctoral Working Station Tianjin Chase Sun Pharmaceutical Co., Ltd. Tianjin China; ^4^ Precision Medicine Division Health‐Biotech (Tianjin) Stem Cell Research Institute Co., Ltd. Tianjin China; ^5^ Jiangsu Institute of Hematology the First Affiliated Hospital of Soochow University Suzhou China

**Keywords:** Aplastic anaemia, genetic alteration, hUC‐MSCs, immunoregulation, proangiogenesis, VCAM‐1

## Abstract

**Objective:**

Longitudinal studies have indicated VCAM‐1^+^ mesenchymal stem/stromal cells (MSCs) as promising resources in regenerative medicine, yet the abundance in gene expression is far from adequate in the advantaged and “discarded” hUC‐MSCs. Thus, high‐efficient preparation and systematic dissection of the signatures and biofunctions of the subpopulation is the prerequisite for large‐scale clinical applications.

**Materials and methods:**

We primarily took advantage of a cytokine‐based programming strategy for large‐scale VCAM‐1^+^ hUC‐MSC generation (III‐MSCs). Thereafter, we conducted multifaceted analyses including cytomorphology, immunophenotype, cell vitality, multilineage differentiation, whole‐genome analysis, tube formation and Matrigel plug assay, lymphocyte activation and differentiation, and systemic transplantation for aplastic anaemia (AA) treatment.

**Results:**

III‐MSCs with high‐proportioned VCAM‐1 expression were obtained by combining IL‐1β, IL‐4 with IFN‐γ, which exhibited comparable immunophenotype with untreated hUC‐MSCs (NT‐MSCs) but revealed multidimensional superiorities both at the cellular and molecular levels. Simultaneously, systemic infusion of III‐MSCs could significantly ameliorate clinicopathological features and finally help facilitate haematopoietic reconstruction and immunoregulation in AA mice.

**Conclusions:**

We have established a high‐efficient procedure for large‐scale generation of III‐MSCs with preferable signatures and efficacy upon aplastic anaemia in mice. Our findings suggested that III‐MSCs were advantageous sources with multifaceted characteristics for regenerative medicine.

## Graphical abstract

Longitudinal studies have indicated the preferable potential of VCAM‐1^+^ mesenchymal stem/stromal cells (MSCs) in regenerative medicine, yet the low abundance is the lingering issue. Herein, we have established a high‐efficient procedure for large‐scale generation of III‐MSCs with preferable signatures and efficacy upon aplastic anaemia in mice.

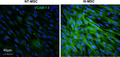

## 1 INTRODUCTION

Mesenchymal stem/stromal cells (MSCs) are acknowledged as a heterogeneous population and the most important stromal cells in the niche for haematopoiesis and coordinate contribution to regenerative medicine.^1^, ^2^, ^3^ For decades, we and other investigators have reported the establishment of MSCs from a various range of adult tissues such as bone marrow, adipose tissue, dental pulp and even human pluripotent stem cells (hPSCs).^2^, ^4^, ^5^, ^6^ Differ from the adult tissue‐derived counterparts, MSCs extracted from perinatal tissues (eg, umbilical cord, placenta) have been proved with preferable characteristics in multifaceted signatures such as juvenility and easy preparation without acquired pollution or ethical risk, and in particular, the hUC‐MSCs with vigorously long‐term reproductive capacity and better cellular pharmaceutical prospects.^2^ Simultaneously, there is a suspicious attitude towards the therapeutic effect and variability in quality in considering the allogeneic cell sources and the probability of genetic variability.^7^, ^8^ For instance, we and Zhang, et al recently reported the multidimensional alterations in efficacy on acute graft‐versus‐host disease and acute liver failure, respectively.^2^, ^9^ Moreover, it is worth noting that functional heterogeneity for MSC‐based cytotherapy has attracted the attention of investigators both in the field of fundamental and translational research, which indicates the urgent need to distinguish alternatively inexhaustible subpopulations of MSCs.^10^, ^11^

To date, limited surface biomarkers have been developed for dissecting the subpopulations with potentially unique bioactivity. Distinguish from the conventionally heterogeneous MSCs, a novel MSCA‐1^+^CD56^+^ subset exhibited predominant differentiation potential towards chondrocytes and pancreatic‐like islets.^12^ Consistently, the enriched CD56^+^ subset from multiclonal BM‐MSCs resulted in enhanced chondrogenesis and bone regeneration.^13^ In recent years, we have originally demonstrated the possibility of generating a highly bioactive subpopulation from the heterogeneous MSCs, especially for high‐quality VCAM‐1^+^ (CD106^+^) hUC‐MSCs.^14^, ^15^ In general, compared with the CD106^‐^ subset, increased proangiogenic potential, preferable immunomodulatory property and enhanced homing capacity of CD106^+^ MSCs were observed, which collectively indicated the promising applications in cellular therapeutics.^14^, ^16^ However, the expression abundance of VCAM‐1 is far from adequate in the advantaged and “discarded” hUC‐MSCs. Meanwhile, the deficiency of systematic and rigorous clarification on the biological and genetic signatures together with efficacy in vivo still hinders their prospects in regenerative medicine. Hence, the preparation and systematic dissection of the signatures and biofunctions of VCAM‐1^+^ subpopulation is the prerequisite for large‐scale clinical applications.

In this study, we have originally established a rapid and high‐efficiency strategy for generating VCAM‐1^+^ III‐MSCs with multidimensional preponderances in signatures, together with highly conserved genome and superior efficacy for AA treatment. Taken together, we concluded that it was feasible to generate large‐scale and homologous III‐MSCs with preferable biological and genetic attributes together with therapeutic effects on aplastic anaemia from the “discarded” UC‐MSCs for regenerative medicine.

## 2 MATERIALS AND METHODS

### 2.1 Cell culture and preconditioning

hUC‐MSCs were cultured for further analyses as we previously described.^17^, ^18^ For III‐MSC induction, the hUC‐MSCs were preconditioned with the indicated cytokines and detailed procedures as listed in Table S1 and Supplementary Procedures.

### 2.2 Flow cytometry analysis

FCM assay was conducted as we recently reported.^2^, ^19^, ^20^ In brief, the cells were washed with 1 × PBS for twice and labelled with fluorescence conjugated antibodies (CD73, CD90, CD105, CD106, CD151, CD31, CD34, CD45 and HLA‐DR; CD4, CD8, IFN‐γ, CD25, CD127, IL‐4, FoxP3 and IL‐17A). The antibodies and detailed procedures were listed in Table S2 and Supplementary Procedures.

### 2.3 Multilineage differentiation analysis

Multilineage differentiation of hUC‐MSCs was performed as we recently described.^2^, ^5^, ^19^, ^20^ Briefly, hUC‐MSCs were treated with adipogenic, osteogenic or chondrogenic (stem cell technologies) differentiation medium for 3 weeks, respectively. Thereafter, cells were stained with Oil Red O, Alizarin Red or Alcian Blue for adipogenic, osteogenic or chondrogenic differentiation, respectively. The primer sequences of the indicated genes and detailed procedures were listed in Table S3 and Supplementary Procedures.

### 2.4 Matrigel plug assay in vivo

The in vivo Matrigel plug assay was performed as we previously described.^15^ 1 × 10^6^ UC‐MSCs were resuspended in 400μl Matrigel and implanted into the dorsal area of 6‐week female nude mice. Mice implanted with Matrigel and 1 × PBS were used as negative controls. 21 days later, mice were euthanized and Matrigel plugs were harvested and fixed in 10% formaldehyde for haematoxylin and eosin (H&E) staining. Micro‐vessels were photographed and calculated under the microscope.

### 2.5 Co‐culture of lymphocytes with hUC‐MSCs

hUC‐MSCs were co‐cultured with CD4^+^ T cells at a ratio of 1:10 as we described before.^2^, ^21^ For Th cell analysis, 100 ng/mL Phorbol‐12‐myristate‐13‐acetate (PMA) (Sigma),1 μg/mL Ionomycin (Sigma) and 2 μmol/L monensin (BD Biosciences) were used to stimulate T cells. For regulatory T (Treg)‐cell differentiation, 5 ng/mL IL‐2 (PeproTech) was added to culture medium for 72 hours.

### 2.6 Aplastic anaemia (AA) model and MSC transplantation

The AA model was conducted as described with several modifications.^22^ In brief, CByB6F1 recipients were pre‐irradiated with 5 Gy of total body irradiation (TBI). Then, lymph node (LN) cells from B6 donor mice were intravenously infused into recipients (2 × 10^6^ LN cells per mouse) within 4‐6 hours. At day 1 and day 4, 1 × 10^6^ NT‐MSCs and III‐MSCs were systemically transplanted into the experimental CByB6F1 mice, respectively. 2 weeks later (from the initiation of the AA model), the mice were euthanized for further analyses. The experiments were approved by the ethical Committee of Institute of Hematology and Blood Diseases Hospital, Chinese Academy of Medical Science and Peking Union Medical College (approval no. KT2019048‐EC‐1).

### 2.7 Statistical analysis

Statistical analysis was performed with Graph Pad Prism 6.0 software as we previously reported.^20^, ^23^, ^24^, ^25^ In brief, we utilized Student's unpaired t test and one‐way ANOVA to analyse the data of two unpaired groups and multiple unpaired groups, respectively. Data were shown as mean ± SEM, and *P* < .05 was considered statistically significant. *P < .05; **P < .01; ***P < .001; ****P < .0001.

## 3 RESULTS

### 3.1 High‐efficient generation of III‐MSCs with preferable multilineage differentiation capacity

Recently, we have originally demonstrated VCAM‐1 as a novel biomarker of MSC subpopulation with unique attributes including superior proangiogenic and immunomodulatory properties.^14^, ^15^ However, the proportion of the VCAM‐1^+^ subset is desperately inadequate for large‐scale application in regenerative medicine.^26^ Simultaneously, the rigorous and systematic evaluation of multidimensional signatures both at the cellular and molecular levels is dauntingly unprocurable.

**FIGURE 1:**
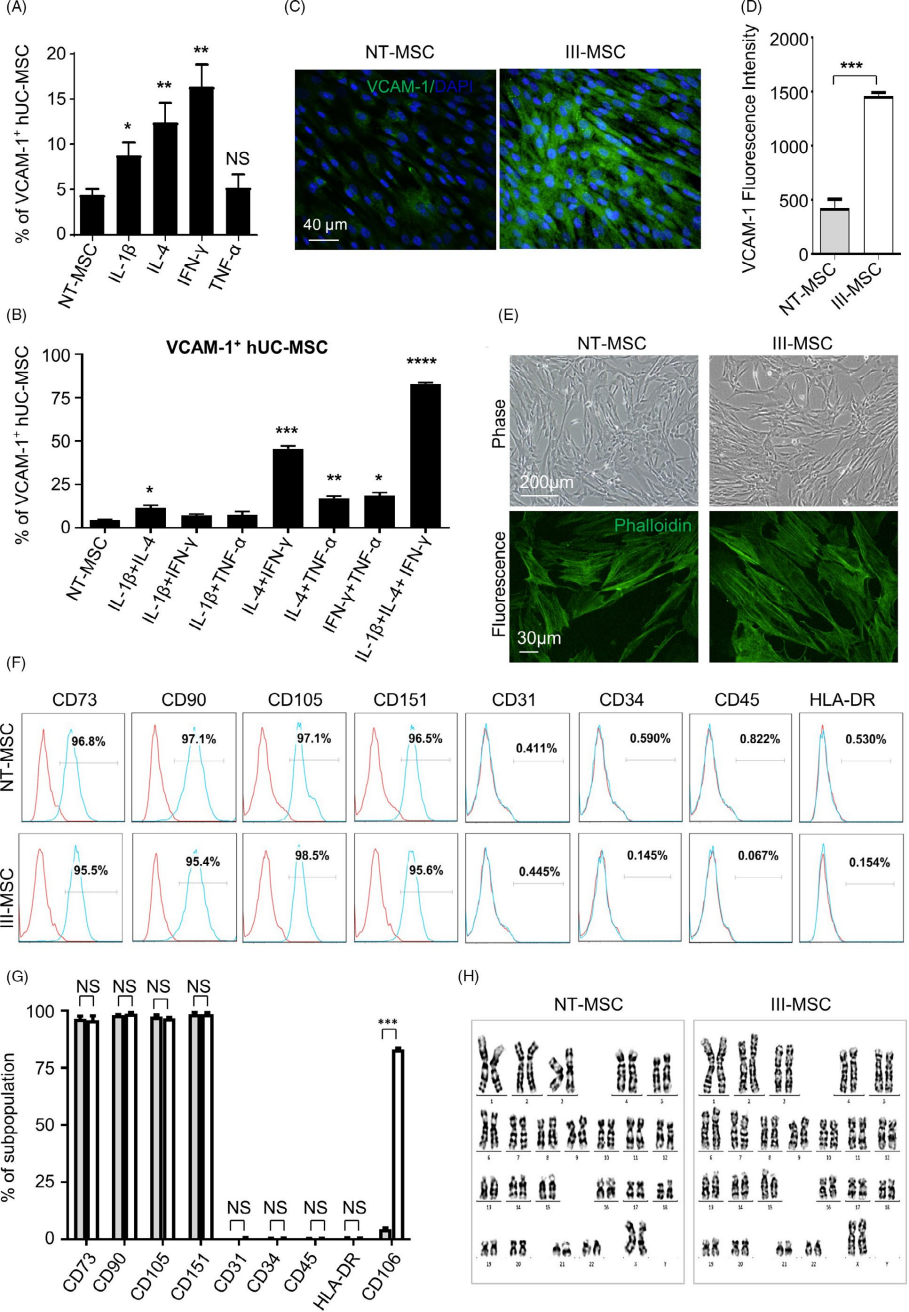
High‐efficient generation of III‐mesenchymal stem/stromal cells (MSCs) with high expression level of VCAM‐1. A and B, FCM analysis of VCAM‐1 expression in hUC‐MSCs with single (A) or combined (B) cytokine treatment. C and D, Immunofluorescent images (C) and Mean fluorescence intensity (MFI) (D) of VCAM‐1 in NT‐ and III‐MSCs (scale bar = 40 μm). E, Immunofluorescent images of Phalloidin in NT‐ and III‐MSCs. F and G, FCM diagram (F) and statistical analysis (G) of surface marker expression in NT‐ and III‐MSCs. H, Karyotype analysis of NT‐ and III‐MSCs. Data were shown as mean ± SEM (n = 3). *P < .05; **P < .01; ***P < .001; ****P < .0001; NS, not significant

For the purpose, we took advantage of a programming strategy to facilitate VCAM‐1^+^ hUC‐MSC generation by utilizing small‐scale cytokine screening as we recently reported with modification.^5^, ^19^ Of them, we found IL‐1β, IL‐4 and IFN‐γ could improve the generation of VCAM‐1^+^ cells, respectively (Figure 1A; Figure S1A‐B). Thereafter, we further verified that the invariably maximum efficiency for inducing VCAM‐1^+^ hUC‐MSCs was 83.00% ± 4.44% by combining the three cytokines (Figure 1B; Figure S1C‐D). To distinguish from the untreated hUC‐MSCs (NT‐MSCs), we denoted the aforementioned hUC‐MSCs with high‐proportioned VCAM‐1^+^ population as III‐MSCs, which were intuitively confirmed by qRT‐PCR detection and immunofluorescent staining (Figure 1C‐1D; Figure S1E). Furthermore, to explore whether IL‐1β, IL‐4 and IFN‐γ treatment might influence the cellular properties including morphology, common biomarker expression and chromosomal stability, we conducted cytoskeletal staining, flow cytometry and karyotypic analysis and preliminarily excluded these possibilities, respectively (Figure 1E‐1H; Figure S1F). Additionally, to illuminate the potential influence of the indicated cytokine addition on the multilineage differentiation capacity, we took advantage of the procedure to differentiate NT‐MSCs and III‐MSCs towards adipocytes, osteoblasts and chondrocytes, respectively. Compared with undifferentiated NC‐MSCs‐ and differentiated NT‐MSC‐derived cells, III‐MSCs exhibited preferable multilineage differentiation potential over NT‐MSCs, which was confirmed by Oil Red O, Alizarin Red and Alcian Blue staining as well as multilineage‐associated markers (Figure S2A‐D). Taken together, we have high‐efficiently and invariably prepared III‐MSCs with enhanced VCAM‐1 expression by an instantaneously three cytokine‐mediated programming strategy.

### 3.2 III‐MSCs showed conserved landscape of gene expression profiling together with certain alterations

**FIGURE 2:**
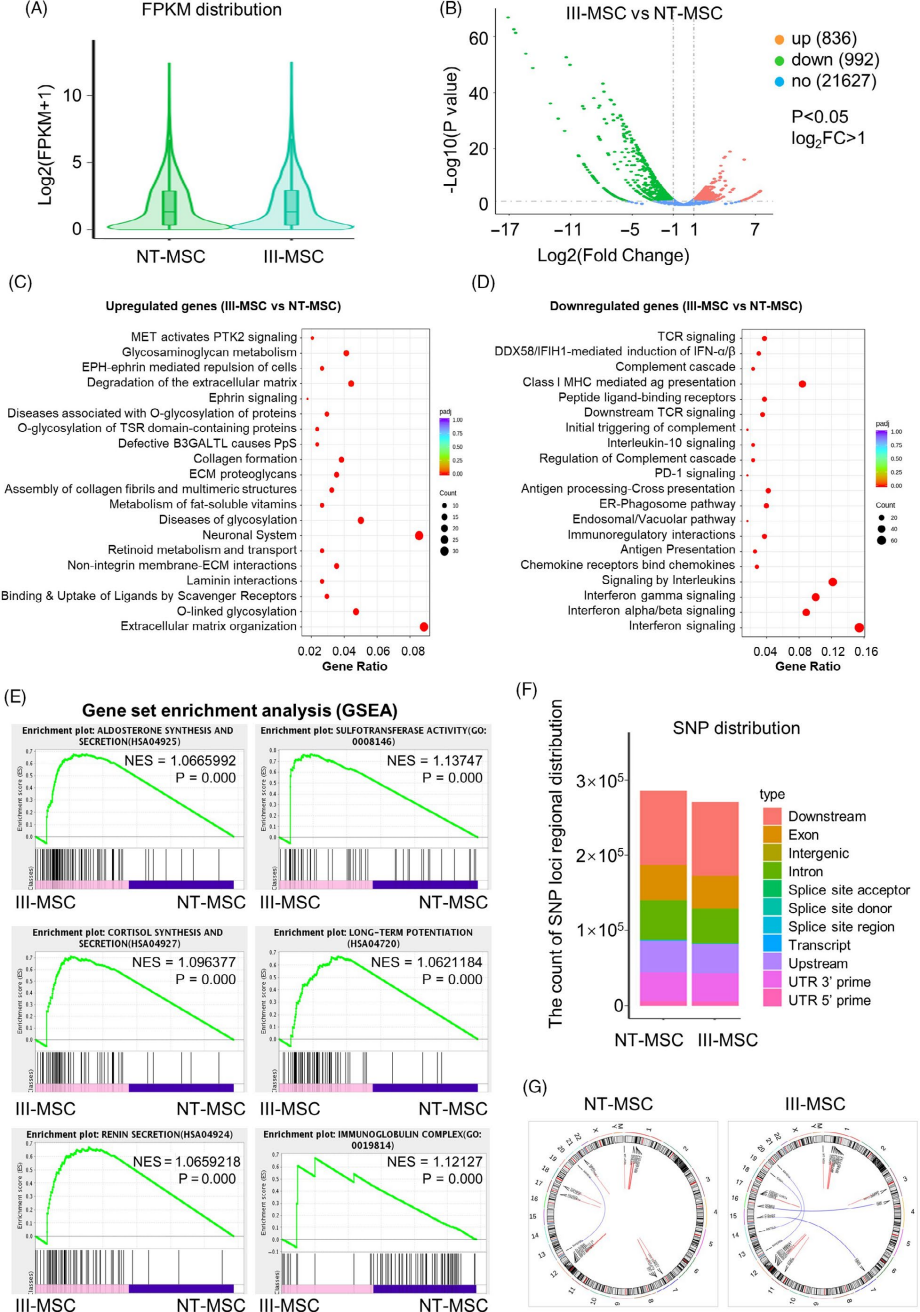
The distribution of differentially expressed genes and mutation spectrum in NT‐mesenchymal stem/stromal cells (MSCs) and III‐MSCs. A, Gene expression distribution of total genes in the genome of NT‐ and III‐MSCs. B, Volcano plot analysis of total genes. C and D, GO analysis of upregulated (C) and downregulated (D) genes (*P* < .05, log_2_ FC > 1). E, GSEA shows enrichment plot of the indicated subsets in NT‐ and III‐MSCs. F and G, Distribution of SNPs (F) and fusion genes (G) in the genome of NT‐ and III‐MSCs

To systematically dissect the potential alterations at molecular level, we turned to gene expression profiling of III‐MSCs and NT‐MSCs. Generally, from the violin illustration and volcano plots, we found the above‐mentioned MSCs showed similarities in total gene expression based on FPKM values whereas 836 and 992 of the 23 455 genes were upregulated and downregulated in III‐MSCs compared with NT‐MSCs, respectively (Figure 2A, B). Furthermore, we noticed the significantly upregulated genes were associated with multiple biological processes including metabolism and extracellular matrix, together with signal pathways (eg, PTK2, Ephrin) (Figure 2C). Conversely, the significantly downregulated genes were mainly involved in immunoregulation‐associated processes such as immunoregulatory interactions, antigen presentation and complement cascades, which were mediated by a variety of signals (eg, TCR, IL‐10, PD‐1, IFN) (Figure 2D). Simultaneously, with the aid of gene set enrichment analysis (GSEA), we further verified that the datasets were representatively enriched in phenotypes such as aldosterone synthesis and secretion, long‐term potentiation and immunoglobulin complex (Figure 2E). Compared with NT‐MSCs, III‐MSCs exhibited similarities in amount of indicated types of SNP loci regional distribution (eg, exon, upstream, UTR 3’ prime) (Figure 2F). Accordingly, somatic variation analysis of gene fusion events (eg, *ABHD12* in NT‐MSCs and III‐MSCs; *TSPAN10* and *MYOCD* in NT‐MSCs; *ICAM1*, *GAK* and *CBX3* in III‐MSCs) by the Circos software intuitionistically reflected their expression and loci regional distribution in the chromosome (Figure 2G). Collectively, these results indicated the multidimensional alterations in a number of gene expression and modification, whereas expression levels of most genes and the types of SNP distributions in the whole‐genome expression profiling were relatively conservative.

### 3.3 III‐MSCs manifested multitudinous similarities with NT‐MSCs in cell vitality

**FIGURE 3:**
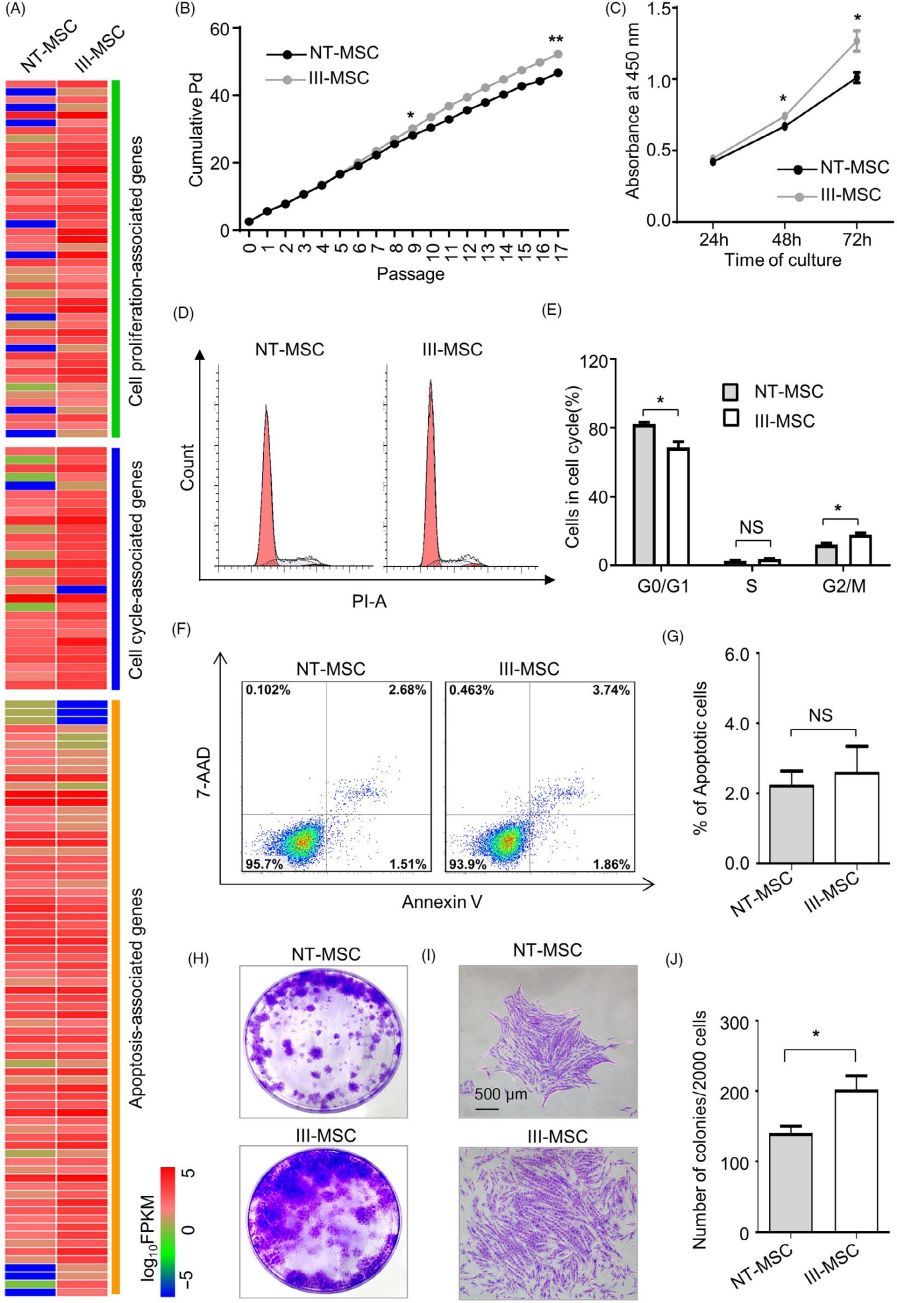
III‐mesenchymal stem/stromal cells (MSCs) with comparable cell vitality but enhanced CFU‐F formation potential. A, Heatmap analysis of gene sets in NT‐ and III‐MSCs. B, Pd assay of NT‐ and III‐MSCs for 17 passages. C, Proliferation assay with CCK‐8 kit. D and E, Distributions of apoptotic population in NT‐ and III‐MSCs as shown by FCM diagram (D) and statistical analysis (E). F and G, The proportion of apoptotic population as shown by FCM diagram (F) and statistical analysis (G). H and I, Images of total CFU‐Fs (H) and representative colonies (I) (scale bar = 500 μm). J, Statistical analysis of CFU‐F numbers. Data were shown as mean ± SEM (n = 3). *P < .05, **P < .01; NS, not significant

Cell vitality and homing capacity are recognized as the fundamental for efficacy in regenerative medicine.^2^, ^9^ As shown by the heatmap diagram, a certain amount of cell proliferation‐associated genes was upregulated in III‐MSCs whereas no distinct tendency in cell cycle‐ and apoptosis‐associated genes (Figure 3A; Table S4). In consist with the gene clustering spectrum, III‐MSCs showed preferable proliferation capacity as confirmed by the cumulative population doubling (Pd) and Pd analyses, respectively (Figure 3B, C). Furthermore, the distributions of sub‐stages of cell cycle and ageing cells with β‐galactosidase staining in III‐MSCs were distinguish from those in NT‐MSCs whereas merely difference was observed in apoptotic cells (Figure 3D‐G, Figure S3A‐D). Consistent with our previous reports on placental chorionic villi‐isolated VCAM‐1^‐^ and VCAM‐1^+^ CV‐MSCs, the III‐MSCs displayed enhanced fibroblast colony forming unit (CFU‐F) formation capacity together with more loose morphology (Figure 3H‐J). Taken together, III‐MSCs exhibited moderate advantages in multifaceted cellular phenotypes, which indicated the advantageous influence of cytokine pretreatment to cell vitality.

### 3.4 III‐MSCs possessed preferable characteristics in migration and angiogenesis over NT‐MSCs

**FIGURE 4:**
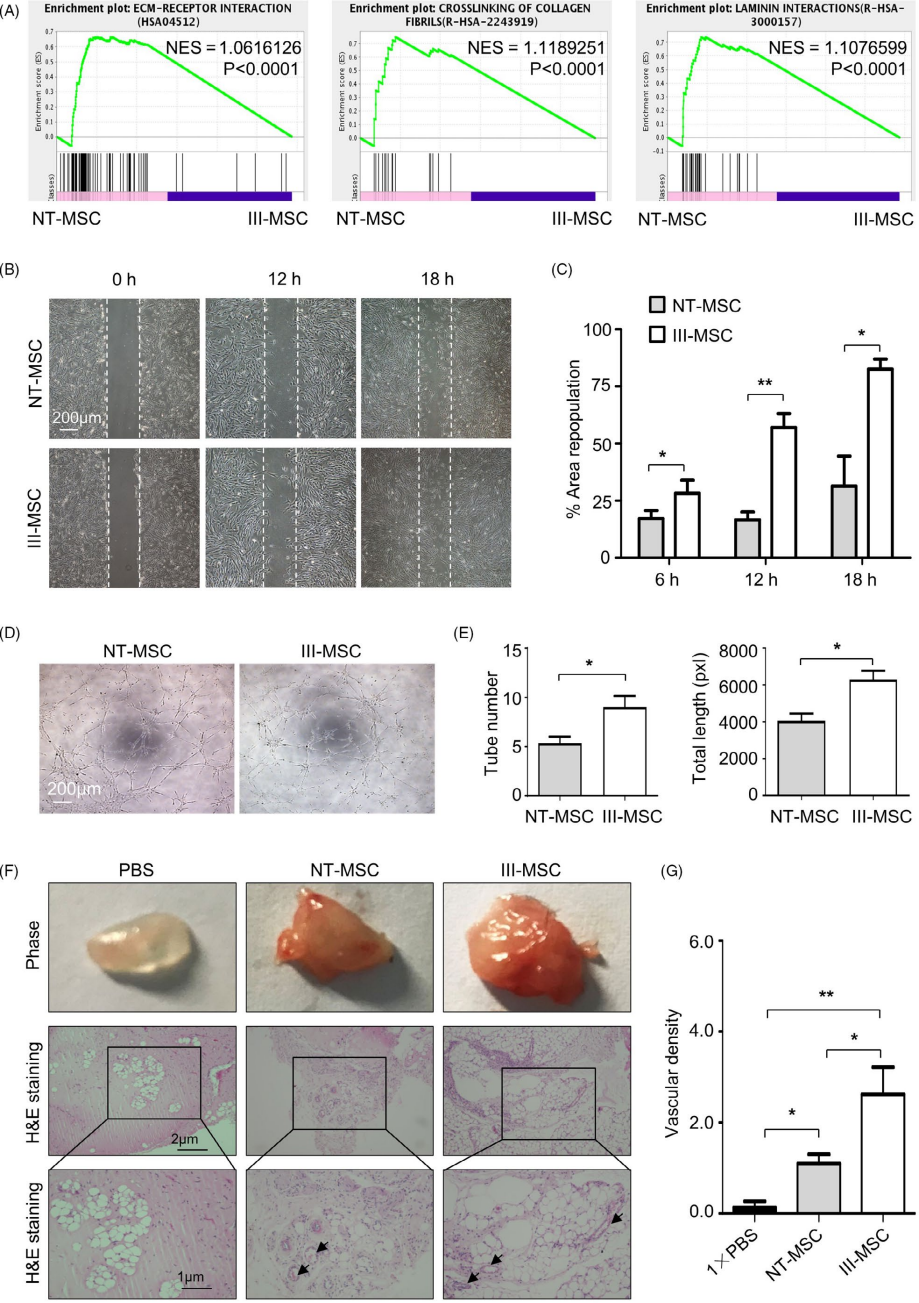
III‐mesenchymal stem/stromal cells (MSCs) exhibited superior characteristics in migration and vasculo‐angiogenic capacity in vitro and in vivo. A GSEA showed enrichment plot of ECM‐receptor interaction and crosslinking of collagen fibrils. B and C, Representative images (B) and statistical analysis (C) of cell migration in NT‐ and III‐MSCs. Scale bar = 200 μm. D and E, Representative images (D) and statistical analysis (E) of in vitro capillary tube‐like structures. Scale bar = 200 μm. F, Matrigel plug assay showed the in vivo vasculo‐angiogenic ability of NT‐ and III‐MSCs. Scale bar = 1 μm or 2 μm. G, Statistical analysis of vascular density of Matrigel plugs with H&E staining. Data were shown as mean ± SEM (n = 3). *P < .05, **P < .01

Therewith, we were curious about whether migration and proangiogenic activities were influenced as well. GSEA analysis intuitively indicated that ECM‐receptor interaction‐, crosslinking collagen fibrils‐ and laminin interactions‐associated gene sets were notably disparate, which were confirmed by assessments on cell migration by wound healing analysis (Figure 4A‐C).

Proangiogenic activity is an advantaged property of VCAM‐1^+^ subpopulation in hUC‐MSCs and hCV‐MSCs.^14^, ^15^ Thus, we conducted the tubular network formation assay in vitro and found III‐MSCs possessed moderate preponderance in spontaneously formed intact tubular structures over NT‐MSCs (Figure 4D, E). Thereafter, with the aid of the well‐established in vivo Matrigel plug angiogenesis assay, we found more macroscopic blood vessels were formed and distributed in the Matrigel plugs of III‐MSCs (Figure 4F, G). Collectively, these data revealed that III‐MSCs had preponderances in migration and proangiogenesis both in vitro and in vivo.

### 3.5 III‐MSCs exhibited superiorities in immunosuppressing proinflammatory cytokine secretion and lymphopoiesis in vitro

**FIGURE 5:**
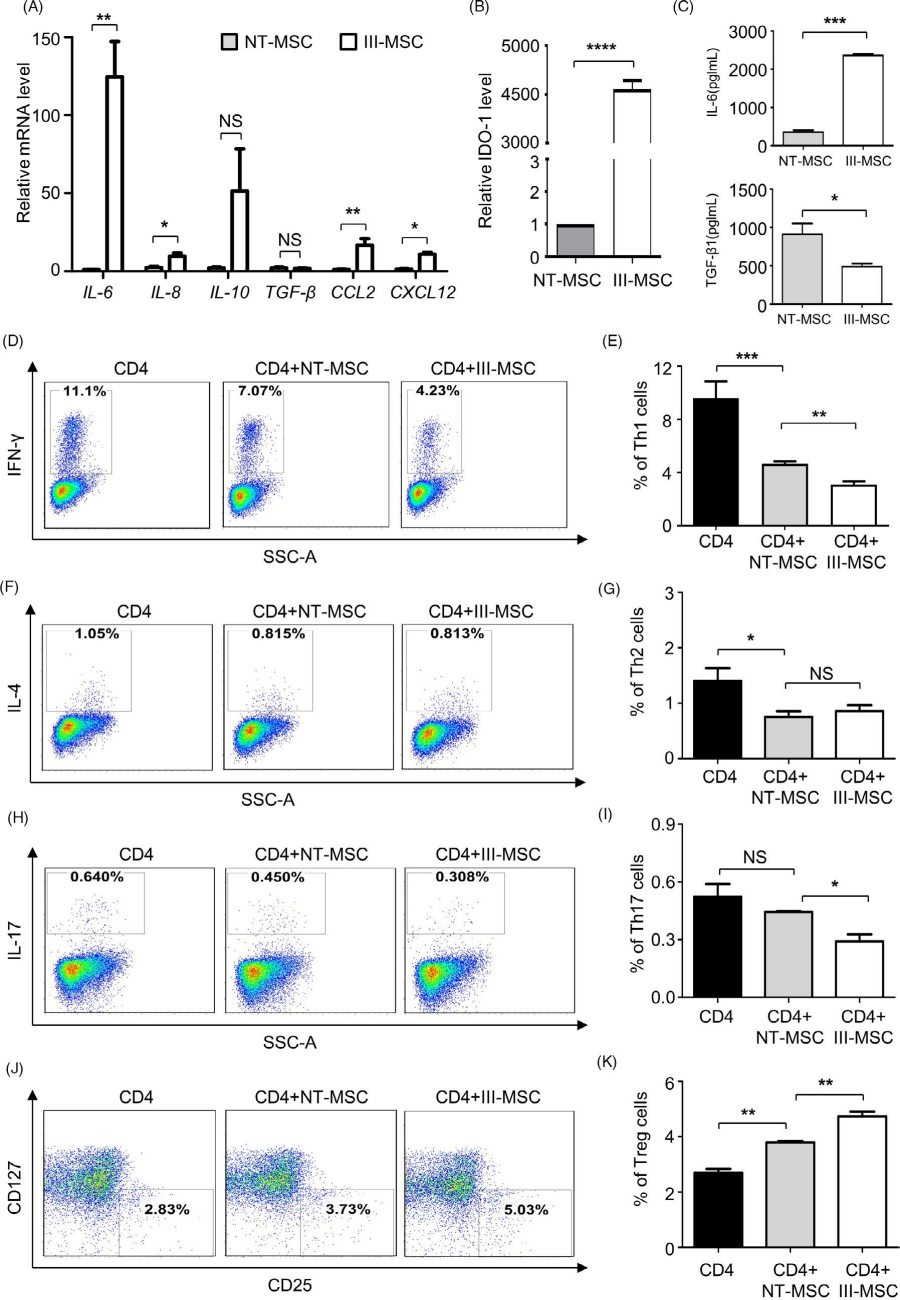
III‐mesenchymal stem/stromal cells (MSCs) displayed preferable immunoregulatory ability over NT‐MSCs in vitro. A and B, qRT‐PCR analysis of multiple immunoregulation‐associated cytokines (A) and *IDO‐1* (B) in NT‐MSCs and III‐MSCs. C, ELISA analysis of secreted cytokines in the supernatant. D‐K, FCM and statistical analyses of the differentiated Th1 (D and E), Th2 (F and G), Th17 (H and I) and Treg (J and K) cells by coculturing CD4^+^ T cells with NT‐ or III‐MSCs. Data were shown as mean ± SEM (n = 3). *P < .05, **P < .01, ***P < .001, ****P < .0001; NS, not significant

To explore the immunosuppressive signature of III‐MSCs, we primarily analysed the expression pattern of inflammatory‐associated cytokines and found the expression levels of representative members of CXC chemokine family (eg, CXCL2) and interleukin family (IL‐6, IL‐8) and IDO‐1 as well as IL‐6 and TGF‐β1 secretion were higher in III‐MSCs (Figure 5A‐C; Table S4). When compared with NT‐MSCs, the inhibitory effect of III‐MSCs on CD4^+^ T lymphocyte activation and differentiation towards Th1 and Th17 cells were enhanced by III‐MSCs, whereas the abundance of Treg subpopulation was increased (Figure 5D‐K). In summary, our data indicated that III‐MSCs exhibited reinforced immunoregulatory properties.

### 3.6 III‐MSCs displayed enhanced ameliorating effect on aplastic anaemia mice compare to NT‐MSCs

**FIGURE 6:**
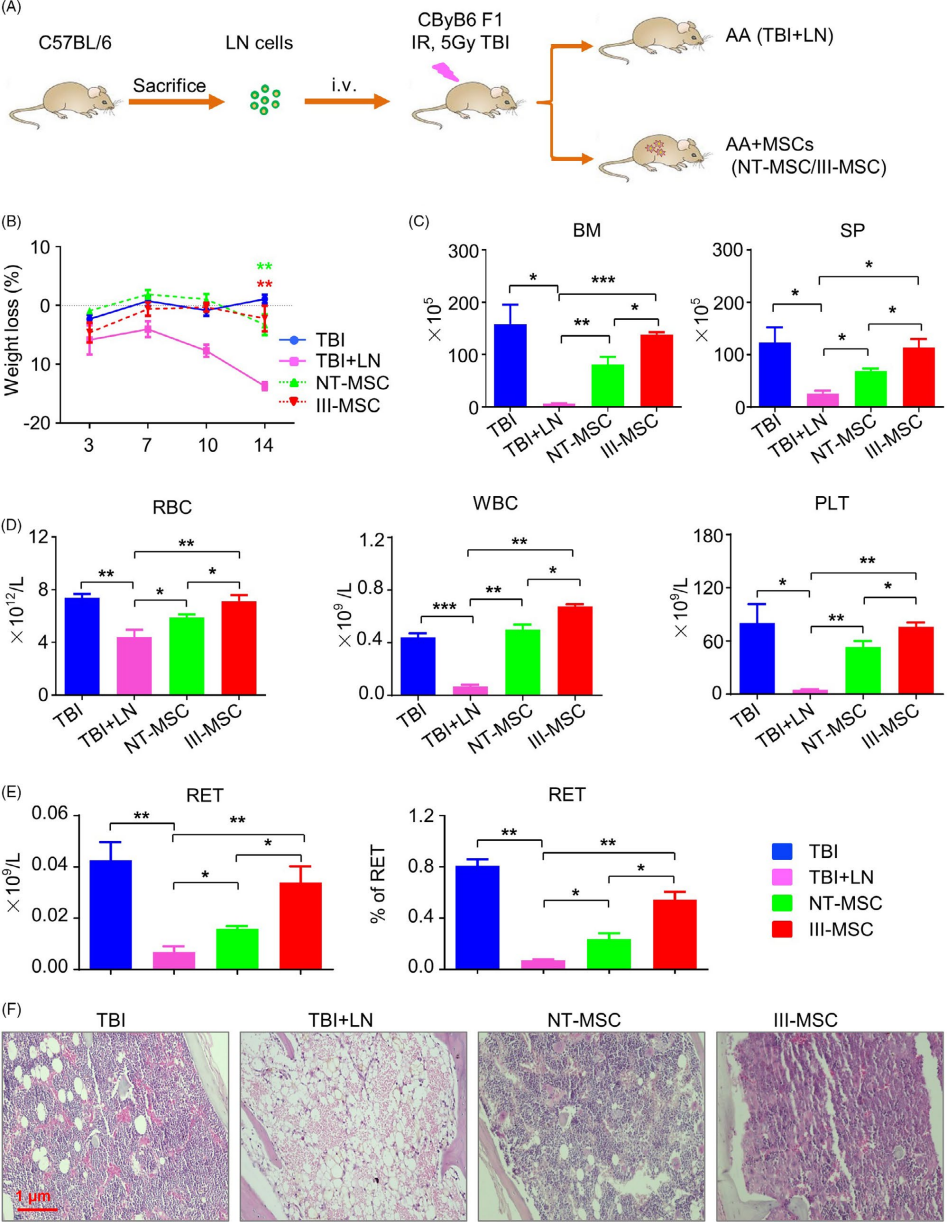
The haemogram and clinicopathologic features of AA mice were alleviated by III‐mesenchymal stem/stromal cell (MSC) transplantation. A, Schematic of the AA mice model. B, Body weight loss of mice (%) in the indicated groups. C, Statistical analysis of nucleated cells and splenocytes in bone marrow (BM) and spleen (SP) of mice. D and E, Statistical analysis of subpopulations in the blood of recipient mice including RBC, WBC (D) and reticulocyte (RET) (E). F, Pathological sections of sterna with H&E staining in the indicated groups. Scale bar = 1 μm. Data were shown as mean ± SEM (n = 5). *P < .05, **P < .01, ***P < .001

Having illuminated the potential superiorities in vivo, we turned to immune‐mediated aplastic anaemia model to investigate the alleviative effect of III‐MSC infusion on pancytopenia and immunodysfunction (Figure 6A). Compared with the recipient mice received total body irradiation (TBI) and lymph node (LN) cell infusion from donor mice (denoted as AA), AA mice with intravenous III‐MSC injection exhibited significantly alleviated body weight loss together with preferable regain of total cell number in bone marrow (BM) and spleen (SP) (Figure 6A‐C). Routine analysis of peripheral blood revealed that the phenomenon of pancytopenia could be further alleviated by MSC transplantation (Figure 6D, E). Consistently, AA mice received III‐MSCs administration displayed preferably ameliorated clinicopathological features as well (Figure 6F).

**FIGURE 7:**
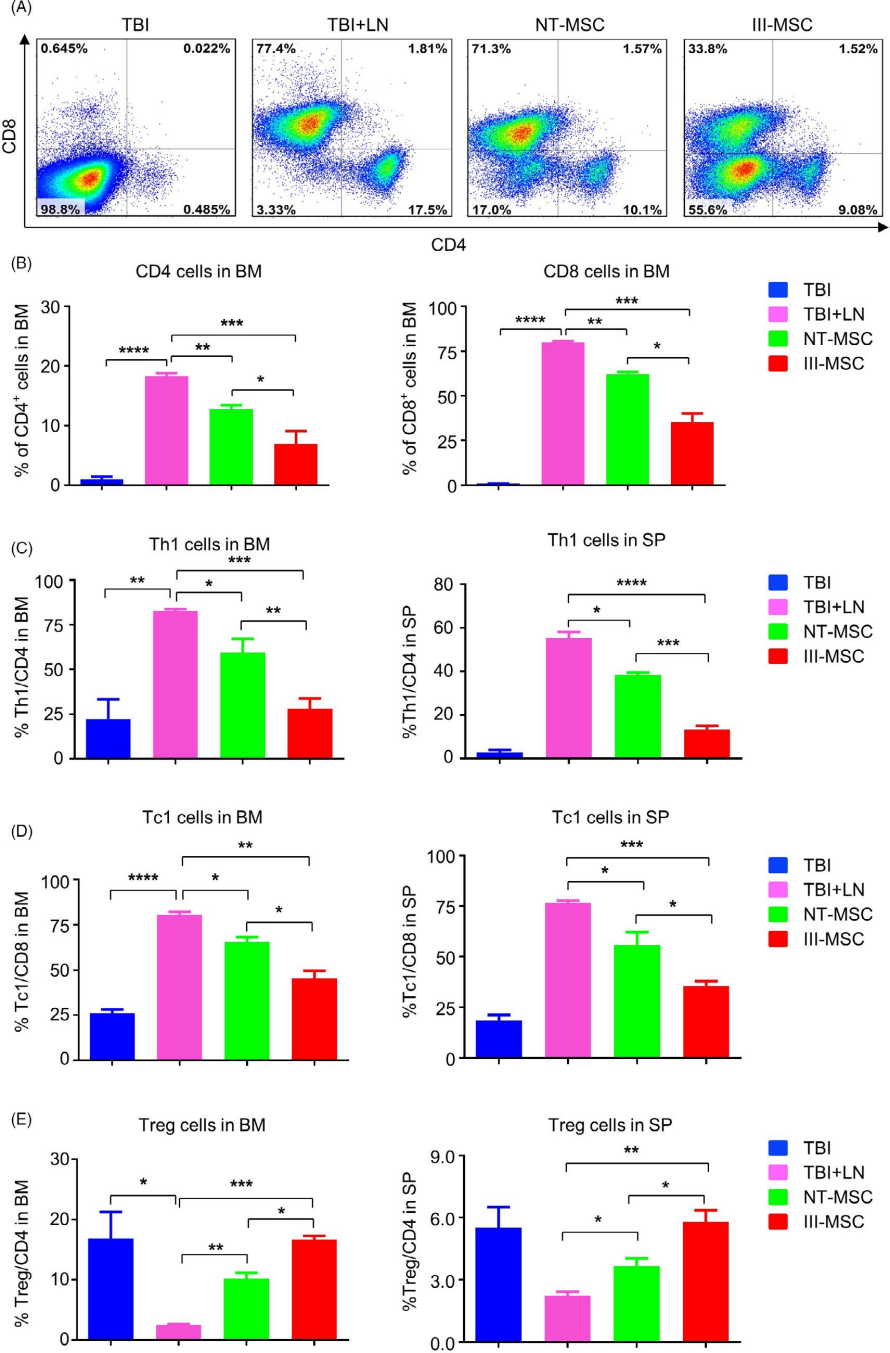
The immunodysfunction of lymphocytes in bone marrow of AA mice was ameliorated by systemic infusion of III‐mesenchymal stem/stromal cells (MSCs). A and B, The distributions of the CD4^+^ and CD8^+^ subpopulations in BM of the indicated mice as shown by the FCM diagram (A) and statistical analysis (B). C and D, Statistical analyses of the CD4^+^ IFN‐γ^+^ Th1 cells (C), CD8^+^ IFN‐γ^+^ Tc1 cells (D), CD4^+^ CD25^+^ FoxP3^+^ Treg cells (E) in the BM and SP of mice, respectively. Data were shown as mean ± SEM (n = 3). *P < .05, **P < .01, ***P < .001, ****P < .0001

To explore the mitigative effect of MSCs to the abnormal hyperimmune state of AA mice, we meticulously analysed the contents of the CD4^+^ and CD8^+^ T lymphocytes in BM and SP, respectively (Figure 7A; Figure S4A). Statistical analysis further intuitively reflected the significantly immunosuppressive effect of III‐MSCs on CD4^+^ and CD8^+^ subpopulations (Figure 7B; Figure S4B, C). Differ from the Treg subpopulation, III‐MSCs could further conformably decrease the proportion of Th1 and Tc1 cells compared with NT‐MSCs in BM and SP (Figure 7C‐E). In conclusion, III‐MSCs exhibited preferable efficacy via simultaneously promoting hematopoietic reconstruction and immunosuppression of AA mice.

## 4 DISCUSSION

Mesenchymal stem/stromal cells are heterogeneous populations with promising application in regenerative medicine and the clinical efficacy largely depends on cell vitality, nevertheless evolution of signatures and biofunctions is largely obscure.^2^ In recent years, we have originally identified VCAM‐1^+^ MSCs from perinatal stem cells with unique proangiogenic and immunoregulatory properties, yet the amount is far from satisfaction. Herein, we have established a high‐efficient programming strategy for VCAM‐1^+^ III‐MSC generation from the most proliferative and youthful hUC‐MSCs without gene editing. Thereafter, we meticulously and systematically dissected the multidimensional alterations of biological and genetic signatures in III‐MSCs, together with the curative effect on AA mice as well. Overall, our studies have provided an alternative procedure for large‐scale VCAM‐1^+^ hUC‐MSC preparation and assessment.

VCAM‐1, also known as CD106, is a transmembrane glycoprotein incipiently identified in endothelial cells, followed by multiple immune cells and stromal cells such as macrophages, dendritic cells and MSCs.^27^ To date, VCAM‐1 has been involved in sensitive MSC‐T lymphocyte interaction and MSC‐mediated immunosuppression and even proangiogenesis.^14^, ^15^, ^28^ For decades, we and other investigators have been assiduously struggling with preparing large‐scale VCAM‐1^+^ MSCs for applications in translational medicine such as high‐density culture condition and stimulation with inflammatory factors, yet the production is far from enough.^29^ Herein, we have established a programming strategy without gene editing for convenient III‐MSC generation and systematical evaluation, which is the prerequisite for large‐scale clinical applications.

State‐of‐the‐art updates towards MSCs prompt the prospects and feasibility for application in disease treatment and health management, especially for recurrent and refractory disorders.^2^, ^30^, ^31^ However, several studies argued that the efficacy of MSCs on rheumatology and intervertebral disc degeneration treatment or heart regeneration was suspicious and even controversial.^7^, ^8^, ^32^ On the basis of analysing the root of the above‐mentioned issue, the cell vitality and homing are acknowledged as the foremost elements need to be taken into consideration.^2^, ^33^, ^34^ On the one hand, MSCs with diverse origins and culture conditions possess disparate signatures and biofunctions. For instance, hUC‐MSCs and hPSC‐MSCs have been demonstrated with preferable property in long‐term in vitro proliferation, together with more robust immunoregulatory capacity.^2^, ^5^, ^19^ However, MSCs at various passages or from different individuals could display alteration in multifaceted signatures and hereditary stability.^2^, ^9^, ^35^ In this study, we also observed multifaceted variations in the efficacy on AA treatment between III‐MSCs and NT‐MSCs. On the other hand, increasingly researches attempt to verify the heterogeneity of MSCs by dissecting the concealed subpopulations, which is also the principal issue for standardizing and guiding therapeutic uses of MSCs.^11^, ^36^

In 2013, we successfully identified the VCAM‐1^+^ subpopulation in MSCs with various origins and confirmed the splendid characteristics in proangiogenesis and immunoregulation by comparing CD106^+^ subpopulation with CD106^‐^ cells.^14^, ^15^ However, the abundance of VCAM‐1 is far from adequate in the advantaged and “discarded” hUC‐MSCs without ethical risk and acquired influence.^14^ Meanwhile, the proportion of VCAM‐1^+^ MSCs in patients with bone marrow failure of acquired AA was sharply declined, which collectively confirmed the negative correlation and potential application with AA as well.^14^

Taken together, our data provided a profound evidence for VCAM‐1^+^ MSCs as a novel and pivotal subpopulation with multidimensional superiorities in signatures and efficacy, together with supplying overwhelmingly new references for explaining the above‐mentioned existing dispute on MSC‐based clinical therapeutics. Above all, even though VCAM‐1^+^ hUC‐MSCs have been high‐efficiently manufactured by cytokine‐based programming, yet there are extensive efforts associated with safety, effectiveness and repeatability need to be done before large‐scale applications in cytotherapy.

## CONFLICT OF INTEREST

The authors declare there is no competing interest and all authors consent to publish the data.

## AUTHOR CONTRIBUTIONS

YW, and LZ: collection and assembly of data, manuscript writing; YC, XR, YG, BS, CL, ZH: collection and assembly of data; YW, XR: MSC transplantation and collection of data; LZ, LZ, and ZH: conception and design, data analysis and interpretation, final approval of the manuscript.

## Supporting information

Supplementary Material

Figure S1

Figure S2

Figure S3

Figure S4

Table S4

## Data Availability

All data generated or analysed during this study are included in this published article and its supplementary information files. Meanwhile, the data sets used and analysed during the current study are also available from the corresponding author on reasonable request.
