# Supplementary material for: High‐efficient generation of VCAM‐1+ mesenchymal stem cells with multidimensional superiorities in signatures and efficacy on aplastic anaemia mice
Source: Cell Prolif. 2020 Jun 29;53(8):e12862. doi: 10.1111/cpr.12862 (PMC7445411; doi:10.1111/cpr.12862)
Supplement: Supplementary file 1 — Supplementary Material [file CPR-53-e12862-s001.docx]

**Supplementary Information**

**High-efficient Generation of VCAM-1^+^ Mesenchymal Stem Cells with Multidimensional Superiorities in Signatures and Efficacy on Aplastic Anemia Mice**

Yimeng Wei^1^**^§^**, Leisheng Zhang^1-4^**^§^**^*^, Ying Chi^1^, Xiang Ren^1^, Yuchen Gao^1^, Baoquan Song^5^, Chengwen Li^1^, Zhibo Han^1^, Lei Zhang^1*^, Zhongchao Han^1,4*^

**Supplementary Information:**

Supplementary Figure Legends for Figure S1-S4;

Supplementary Tables: Table S1-S3;

Supplementary Table S4: Gene expression profiling of NT-MSC and III-MSC;

Supplementary Procedures;

Supplementary References.

**Supplementary Figure Legends**

**Figure S1 The expression levels of VCAM-1 in hUC-MSCs with diverse ways of preconditioning.**

**a** The proportions of VCAM-1^+^ population in 3 independent hUC-MSCs with or without single cytokine preconditioning (mean±SEM, n=3). **b** Representative FCM diagrams of the proportions of VCAM-1^+^ population in hUC-MSCs with or without single cytokine preconditioning. **c** The proportions of VCAM-1^+^ population in hUC-MSCs with or without combination of cytokine preconditioning (mean±SEM, n=3). **d** Representative FCM diagrams of the proportions of VCAM-1^+^ population in hUC-MSCs with or without combination of cytokines preconditioning. **e** Relative mRNA expression levels of VCAM-1 in NT- MSC and III-MSC. Data were shown as mean±SEM (n=3). ***P<0.001. **f** Quantitative analysis of pluripotency-associated genes including *POU5F1*, *SOX2* and *NANOG* in NT-MSC and III-MSC. Data were shown as mean±SEM (n=3). *P<0.05; NS, not significant.

**Figure S2 III-MSCs showed enhanced multi-lineage differentiation potential over** **NT-MSCs.**

**a** Oil Red O staining (upper panel), Alizarin Red staining (middle panel), Alcian Blue staining (bottom panel) of the indicated MSCs towards adipocytes. Scale bar=100 μm. **b** qRT-PCR analysis of adipogenic markers of the indicated MSCs towards adipocytes. **c** qRT-PCR analysis of the osteogenic markers of the indicated MSCs towards osteoblasts. **d** qRT-PCR analysis of chondrogenic markers of the indicated MSCs towards chondrocytes. Data were shown as mean ± SEM (n=3). *P < 0.05, **P<0.01.

**Figure S3 Identification of apoptotic and ageing cells in NT-MSCs and III-MSCs**

**a-b** The distributions of the Annexin V^+^ and 7-AAD^+^ apoptotic subpopulations in the indicated NT-MSCs and III-MSCs as shown by the FCM diagram (**a**) and statistical analysis (**b**). Data were shown as Mean±SEM (N=3). NS, not significant. **c** The senescence-associated β-galactosidase assay upon NT-MSCs and III-MSCs. The ageing cells were indicated with the black arrows. Scale bar=200 μm. **d** The fold change of ageing cells with β-galactosidase staining (relative to the III-MSCs group, Mean ± SEM). *P<0.05.

**Figure S4 The immunodysfunction of lymphocytes in spleen of AA mice was ameliorated by systemic infusion of III-MSCs.**

**a-c** The distributions of the CD4^+^ and CD8^+^ subpopulations in spleen of the indicated mice (TBI, TBI+LN, NT-MSC, III-MSC) were shown by the FCM diagram (**a**) and statistical analysis (mean±SEM, n=5) (**b-c**). **P<0.01; NS, not significant. Data were shown as mean±SEM (n=3). *P<0.05, **P<0.01.

**Supplementary Tables**

**Table S1 Chemical compounds and ELISA kit in this study.**

Chemical compounds and ELISA kit

| Reagent | Cat.NO. | Source |
| --- | --- | --- |
| Human bFGF | AF-100-18B | PeproTech |
| Human EGF | AF-100-15 | PeproTech |
| Human IL-1β | 200-01B | PeproTech |
| Human IL-4 | 200-04 | PeproTech |
| Human IFN-γ | 300-02-20 | PeproTech |
| Human TNF-α | AF-300-01A | PeperoTech |
| Human IL-2 | 200-02-10 | PeproTech |
| Phorbol 12-Myristate 13-Acetate | 524400 | Sigma |
| Ionomycin calcium salt from Streptomyces conglobatus | I0634 | Sigma |
| Protein Transport Inhibitor (Containing Monensin) | 554724 | BD |
| Human IL-6 ELISA kit | EHC007 | Neobioscience |
| Human TGF-β1 ELISA kit | EHC107b | Neobioscience |

**Table S2. Antibodies used in this study.**

Antibodies for flow cytometry assay and immunofluorescent staining.

| Antibody | Cat.NO. | Source |
| --- | --- | --- |
| Anti-CD73-PE | 550257 | BD Pharmingen |
| Anti-CD90-FITC | 561969 | BD Pharmingen |
| Anti-CD105-PE | 560839 | BD Pharmingen |
| Anti-CD151-PE | 556057 | BD Pharmingen |
| Anti-CD31-PE | 560975 | BD Pharmigen |
| Anti-CD34-PE | 560941 | BD Pharmingen |
| Anti-CD45-PE | 560975 | BD Pharmingen |
| Anti-HLA-DR-FITC | 562008 | BD Pharmingen |
| Anti-CD4-FITC | 100405 | Biolegend |
| Anti-CD8a-PerCP-Cy5.5 | 100731 | Biolegend |
| Anti-CD25-APC | 102011 | Biolegend |
| Anti-IFN-γ-APC | 505809 | Biolegend |
| Anti-IL-4-PE | 504105 | Biolegend |
| Anti-IL-17a-PE-Cy7 | 506921 | Biolegend |
| Anti-FoxP3-PE | 126403 | Biolegend |
| Human Th1/Th2/Th17 Phenotyping Kit | 560751 | BD Pharmingen |
| Human CD4 Microbeads, | 130-045-101 | Mitenyi Biotec |
| Rabbit anti-VCAM-1 antibody | ab134047 | Abcam |
| 488 donkey anti-rabbit IgG | R37118 | Invitrogen |

**Table S3. Primers used in this study.**

Real-time PCR primer sequences.

| Gene | Forward Primer | Reserve Primer |
| --- | --- | --- |
| *ACTIN* | CTCTTCCAGCCTTCCTTCCT | AGCACTGTGTGTTGGCGTACAG |
| *POU5F1* | CTTGAATCCCGAATGGAAAGGG | GTGTATATCCCAGGGTGATCCTC |
| *SOX2* | GCCGAGTGGAAACTTTTGTCG | GGCAGCGTGTACTTATCCTTCT |
| *NANOG* | TTTGTGGGCCTGAAGAAAACT | AGGGCTGTCCTGAATAAGCAG |
| *ADIPOQ* | TGGTCCTAAGGGAGACATCG | TGGAATTTACCAGTGGAGCC |
| *PPAR-γ* | GCTGGCCTCCTTGATGAATA | TGTCTTCAATGGGCTTCACA |
| *RUNX2* | CTCACTACCACACCTACCTG | TCAATATGGTCGCCAAACAGATTC |
| *BGLAP* | GGCGCTACCTGTATCAATGG | TCAGCCAACTCGTCACAGTC |
| *ACAN* | CCCCTGCTATTTCATCGACCC | GACACACGGCTCCACTTGAT |
| *SOX9* | AATGGAGCAGCGAAATCAAC | CAGAGAGATTTAGCACACTGATC |
| *VCAM-1* | GGGAAGATGGTCGTGATCCTT | TCTGGGGTGGTCTCGATTTTA |
| *IL-6* | ACTCACCTCTTCAGAACGAATTG | CCATCTTTGGAAGGTTCAGGTTG |
| *IL-8* | CTGCGCCAACACAGAAATTA | TGAATTCTCAGCCCTCTTCAA |
| *IL-10* | TCACATGCGCCTTGATGTCTG | GATGTCAAACTCACTCATGGCT |
| *TGF-β* | GGCCAGATCCTGTCCAAGC | GTGGGTTTCCACCATTAGCAC |
| *IDO1* | AGACTGCTGGTGGAGGACATG | AAAGGACAAACTCACGGACTG |
| *COX-2* | ACTCTGGCTAGACAGCGTAA | ACCGTAGATGCTCAGGGAC |
| *CCL2* | CAGCCAGATGCAATCAATGCC | TGGAATCCTGAACCCACTTCT |
| *CXCL12* | ATTCTCAACACTCCAAACTGTGC | ACTTTAGCTTCGGGTCAATGC |

**Supplementary Procedures**

**Cell culture and preconditioning**

hUC-MSCs at passage 3~8 were cultured and used for *in vitro* and *in vivo* analyses as we previously described[^1^](#_ENREF_1). In details, hUC-MSCs were maintained in DMEM/F12 basal medium (Hyclone), supplemented with 10% fetal bovine serum (Gibco), 1% penicillin-streptomycin (Gibco), 1% L-glutamine (Gibco), 10 ng/mL EGF (PeproTech) and 2 ng/mL bFGF (PeproTech). For preconditioning, hUC-MSCs were seeded in 6-well plates at a density of 5×10^5^ cells/ml for 24 h, then 10 ng/ml recombinant human IL-1β (PeproTech), 10 ng/ml recombinant human IL-4 (PeproTech) and 20 ng/ml recombinant human IFN-γ (PeproTech) were added separately or in combination for another 48h at 37 ℃, 5% CO_2_. The indicated cytokines were listed in Supplementary Information: Supplementary Table S1.

**Flow cytometry analysis**

Flow cytometry assay of the immunophenotype of hUC-MSCs was conducted as we recently reported[^2-4^](#_ENREF_2). In brief, the NT-MSCs and III-MSCs were labeled with fluorescence conjugated antibodies against CD73, CD90, CD105, CD106, CD151, CD31, CD34, CD45 and HLA-DR. Isotype-matched antibodies were used as negative control. For Th cells, CD4^+^ T cells were analyzed with human Th1/Th2/Th17 Phenotyping Kit (BD Biosciences). For Treg cells, CD4^+^ T cells were labeled with fluorescence conjugated antibodies against CD4, CD25 and CD127. For bone marrow nucleated cells and splenocytes in mice, cells were labeled with fluorescence conjugated antibodies against CD4, CD8, IFN-γ, CD25, IL-4, FoxP3 and IL-17A. The indicated antibodies were listed in Supplementary Information: Supplementary Table S2.

**Immunofluorescent staining**

The immunofluorescent assay was performed as we recently described with several modifications[^3^](#_ENREF_3)^,^[^5^](#_ENREF_5). Briefly, hUC-MSCs were seeded into a confocal dish pre-coated with rat tail collagen. After preconditioning for 48 h, MSCs were washed with 1×PBS for twice and fixed for 20 min with 4% formaldehyde at room temperature (RT). Then, the cells were blocked with 5% BSA for 1 h and permeabilizated with 0.3% Triton^TM^ X-100 for 15 min. Thereafter, MSCs were labeled with rabbit-anti-human CD106 and donkey-anti-rabbit Alexa Fluor 488-conjugated secondary antibody, respectively. The nucleus was stained with DAPI. Finally, the labeled cells were observed and photographed with an Ultra VIEW VOX spinning disk confocal microscope (Perkinelmer). The indicated antibodies were listed in Supplementary Information: Supplementary Table S2.

**Cell proliferation assay**

The hUC-MSC proliferation was assessed with the Cell Counting Kit 8 (Dojindo) according to the manufacturer’s instructions at the indicated time points (24 h, 48 h and 72 h) under absorbance at 450 nm. Population doubling (Pd) was calculated according to the formula: PdN=logN/N_0_×3.31 and PdT=(t-t_0_) × log2/log(N-N_0_). (PdN, population doubling number; PdT, population doubling time; N_0_ is the initial number of seeded cells, N is the number of harvested cells and t-t_0_ is time of cell culture). Each experiment was performed in triplicate.

**CFU-F assay**

The CFU-F assay was conducted as we previously reported with several modifications[^6^](#_ENREF_6). In brief, hUC-MSCs were seeded into 5 cm dishes at a density of 2000 cells per dish and culture medium was replaced every 3 days. After 14 days, MSCs were fixed with 4% paraformaldehyde (PFA) and stained with 0.5% crystal violet. The colonies with more than 30 cells were calculated.

**Ageing-associated β-Galactosidase assay**

To assess the ageing of the indicated NT-MSCs and III-MSCs, we conducted senescence-associated β-galactosidase assay as we recently reported[^2^](#_ENREF_2). Briefly, the NT-MSCs and III-MSCs were seeded in MSC culture medium in 6-well plates for 24 h. Then, we took advantage of the senescence β-galactosidase staining kit (Cell Signaling Technology) to analyze the intensity of senescence according to the manufacturer’s instructions. After incubation with the β-Galactosidase Staining Solution, the cells were dried and observed under an Olympus DP71 microscope (Tokyo, Japan).

**Adipogenic, osteogenic and chondrogenic differentiation**

The multi-lineage differentiation analysis of hUC-MSCs was performed as we recently described[^2^](#_ENREF_2)^,^[^3^](#_ENREF_3). In brief, hUC-MSCs were seeded in 12-well plates at a density of 2×10^4^/well. When cells reached 80% confluence, cell culture medium was changed into adipogenic (Human Umbilical Cord Mesenchymal Stem Cell Adipogenic Differentiation Kit, Cyagen), osteogenic (Human Umbilical Cord Mesenchymal Stem Cell Osteogenic Differentiation Kit, Cyagen) and chondrogenic (MesenCult-ACF Chondrogenic Differentiation Kit, Stem Cell Technologies) differentiation medium, respectively. The differentiation medium was changed every 3 days. 21 days later, cells were stained with Oil Red O, Alizarin Red, or Alcian Blue for adipogenic, osteogenic or chondrogenic differentiation, respectively.

**cDNA synthesis by RT-PCR**

The cDNA was synthesized by conducting reverse transcription polymerase chain reaction (RT-PCR) as we previously reported[^3-6^](#_ENREF_3). Briefly, total RNA was extracted by utilizing TRIzol reagent (ThermoFisher) and cDNA was synthesized by TransScript Fly First-Strand cDNA Synthesis SuperMix (Transgen Biotech, China) according to the manufacturer’s instructions.

**Quantitative real-time PCR (qRT-PCR)**

qRT-PCR assay was performed as we described before with several modifications[^2^](#_ENREF_2)^,^[^7^](#_ENREF_7). qRT-PCR was performed with the SYBR™ Green PCR Master Mix (Applied Biosystems) in the QuantStudio 6 Flex Real-Time PCR System (Applied Biosystems). The expression levels of genes were calculated with the 2^–ΔΔCT^ method. ACTIN was used as an internal control. The sequences of the indicated genes were listed in Supplementary Information: Supplementary Table S3.

**Scratch wound healing assay**

A scratch wound was generated with a pipette tip across the center of each well when MSCs reached 80% confluence as we described recently[^8^](#_ENREF_8). After scratching, cells were washed with 1×PBS for twice, and culture medium was replaced by DMEM-F12 supplemented with 2% FBS. Cell migration was observed and photographed at different time points, and the gap area was quantitatively evaluated by Image J software and calculated with the following formula: % of area repopulation = 1- clear area of t_n_/clear area of t_0_ (t_0_ is the initial time point, t_n_ is the end time point).

**Tube formation assay *in vitro***

Tube formation assay was conducted as we previously described[^9^](#_ENREF_9). In brief, hUC-MSCs were seeded into Matrigel-coated 48-well plates at a density of 2×10^4^ cells per well and photographs were taken at the indicated time points. Image J software was used to measure tube numbers and total length of capillary-like structures.

**Co-culture of lymphocytes with hUC-MSCs**

Ficoll density gradient centrifugation was performed to isolate peripheral blood mononuclear cells (PBMCs) from healthy donors as we reported[^8^](#_ENREF_8)^,^[^10^](#_ENREF_10). Purification of CD4^+^ T cells was isolated with relevant magnetic MicroBead kits (Miltenyi Biotec) from PBMCs according to the manufacturer’s instructions. Then, hUC-MSCs and CD4^+^ T cells were co-cultured at a ratio of 1:10 in RPMI-1640 basal medium containing 10% FBS. For T helper (Th) cell analysis, 100 ng/ml Phorbol-12-myristate-13-acetate (PMA) (Sigma) ,1 μg/ml **Ionomycin (Sigma) and 2 μM monensin (BD Biosciences) were used to stimulate T cells. For regulatory T (Treg) cell differentiation, 5 ng/ml IL-2 (PeproTech) was added to culture medium for 72 h.**

**Aplastic anemia (AA) model and MSC transplantation**

CByB6F1 mice are the progeny of C57BL/6 and BALB/c, which are the typical mouse strains for aplastic anemia model construction as we have recently reported^8^. 8-week male C57BL/6 and CByB6F1 mice were purchased from the Laboratory Animal Center of the Institute of Hematology & Blood Diseases Hospital, Chinese Academy of Medical Sciences and Peking Union Medical College. All animal studies were approved by the Peking Union Medical College Institutional Animal Care and Use Committee (license no. SCXK & SYXK 2005-0001, Tianjin).

**Supplementary References**

1. Lu L, Liu Y, Yang S, et al. Isolation and characterization of human umbilical cord mesenchymal stem cells with hematopoiesis-supportive function and other potentials. *Haematologica.* 2006;91(8):1017-1026.

2. Zhao Q, Zhang L, Wei Y, et al. Systematic comparison of hUC-MSCs at various passages reveals the variations of signatures and therapeutic effect on acute graft-versus-host disease. *Stem Cell Res Ther.* 2019;10(1):354.

3. Wei Y, Hou H, Zhang L, et al. JNKi- and DAC-programmed mesenchymal stem/stromal cells from hESCs facilitate hematopoiesis and alleviate hind limb ischemia. *Stem Cell Res Ther.* 2019;10(1):186.

4. Zhang X, Yang Y, Zhang L, et al. Mesenchymal stromal cells as vehicles of tetravalent bispecific Tandab (CD3/CD19) for the treatment of B cell lymphoma combined with IDO pathway inhibitor D-1-methyl-tryptophan. *J Hematol Oncol.* 2017;10(1):56.

5. Wu Q, Zhang L, Su P, et al. MSX2 mediates entry of human pluripotent stem cells into mesendoderm by simultaneously suppressing SOX2 and activating NODAL signaling. *Cell Res.* 2015;25(12):1314-1332.

6. Zhang L, Wang H, Liu C, et al. MSX2 Initiates and Accelerates Mesenchymal Stem/Stromal Cell Specification of hPSCs by Regulating TWIST1 and PRAME. *Stem Cell Reports.* 2018;11(2):497-513.

7. El Moshy S, Radwan IA, Rady D, et al. Dental Stem Cell-Derived Secretome/Conditioned Medium: The Future for Regenerative Therapeutic Applications. *Stem Cells Int.* 2020;2020:7593402.

8. Huo J, Zhang L, Ren X, et al. Multifaceted characterization of the signatures and efficacy of mesenchymal stem/stromal cells in acquired aplastic anemia. *Stem Cell Res Ther.* 2020;11(1):59.

9. Du W, Li X, Chi Y, et al. VCAM-1+ placenta chorionic villi-derived mesenchymal stem cells display potent pro-angiogenic activity. *Stem Cell Res Ther.* 2016;7:49.

10. Zhang W, Liu C, Wu D, et al. Decitabine improves platelet recovery by down-regulating IL-8 level in MDS/AML patients with thrombocytopenia. *Blood Cells Mol Dis.* 2019;76:66-71.
