# Supplementary figures and images for: High‐efficient generation of VCAM‐1+ mesenchymal stem cells with multidimensional superiorities in signatures and efficacy on aplastic anaemia mice
Source: Cell Prolif. 2020 Jun 29;53(8):e12862. doi: 10.1111/cpr.12862 (PMC7445411; doi:10.1111/cpr.12862)

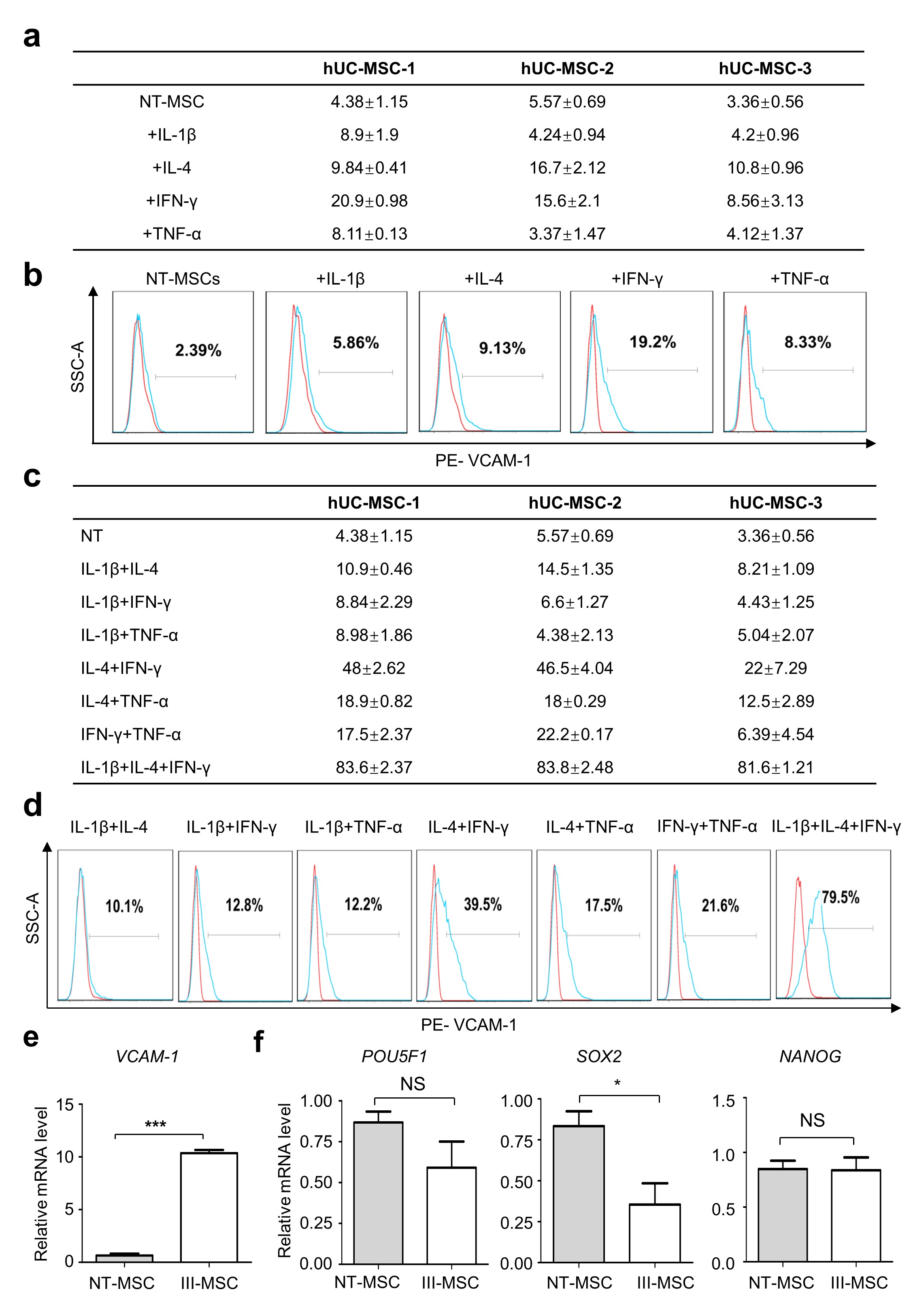

Supplement: Supplementary file 2 — Figure S1 [file CPR-53-e12862-s002.jpg]

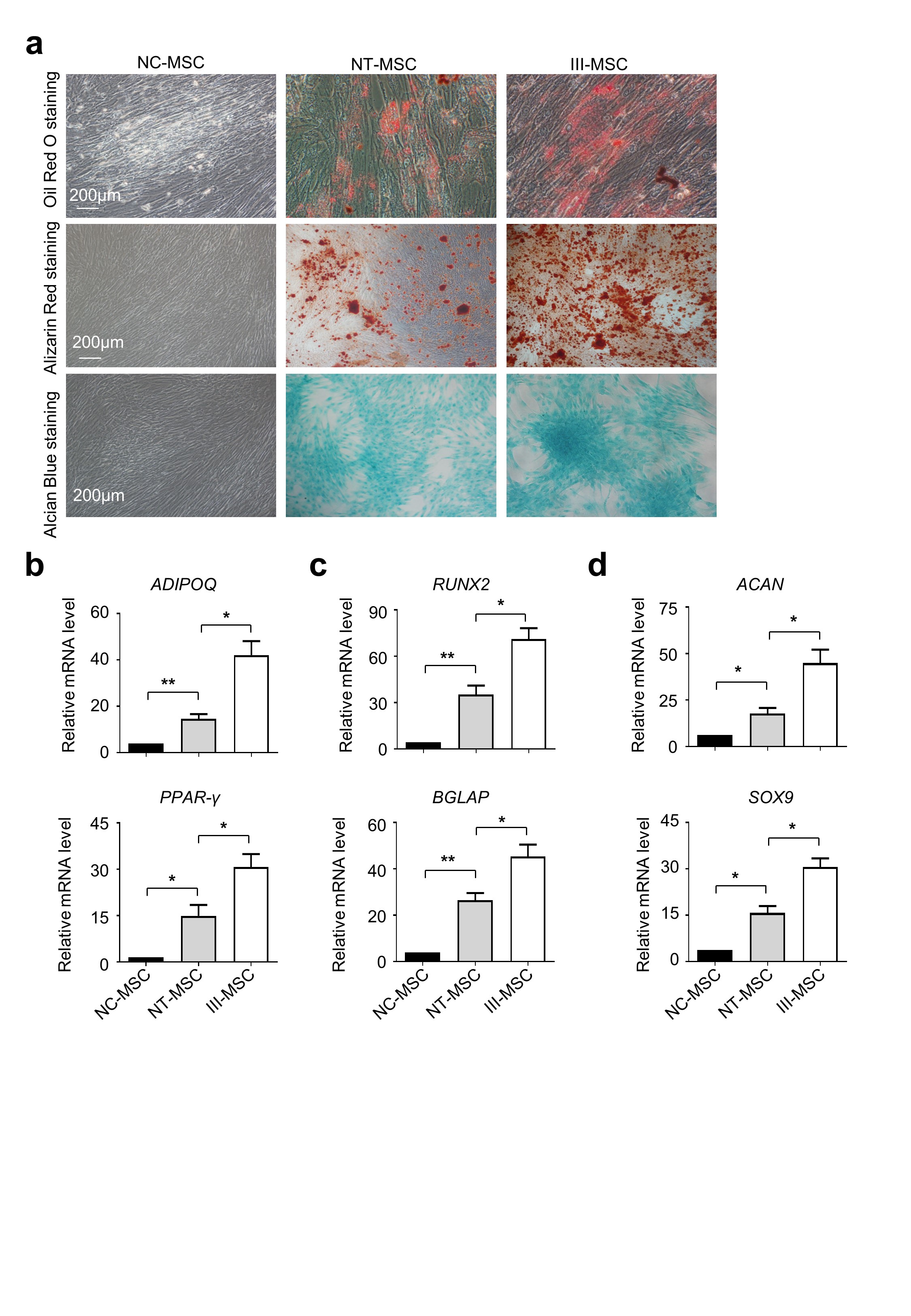

Supplement: Supplementary file 3 — Figure S2 [file CPR-53-e12862-s003.jpg]

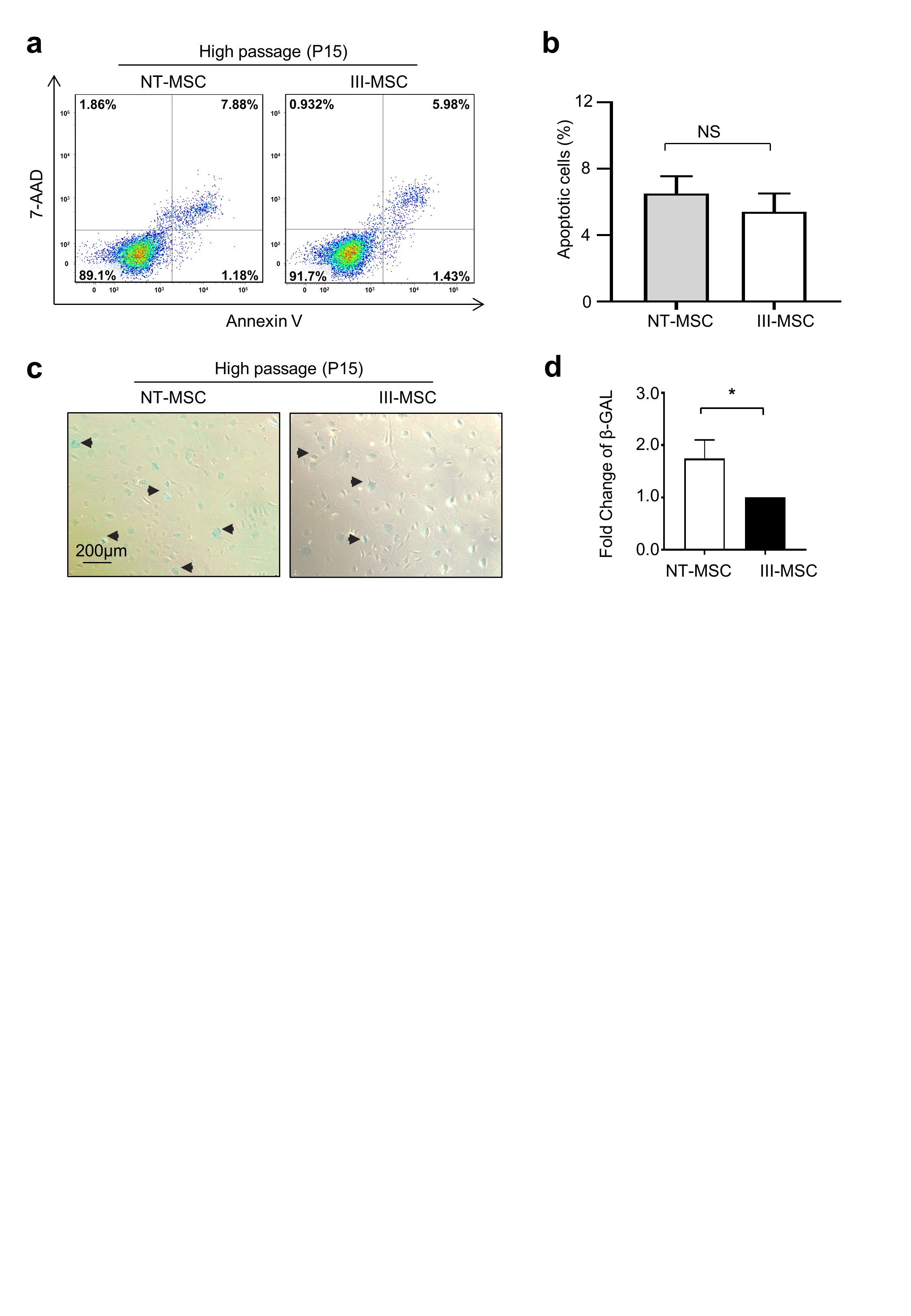

Supplement: Supplementary file 4 — Figure S3 [file CPR-53-e12862-s004.jpg]

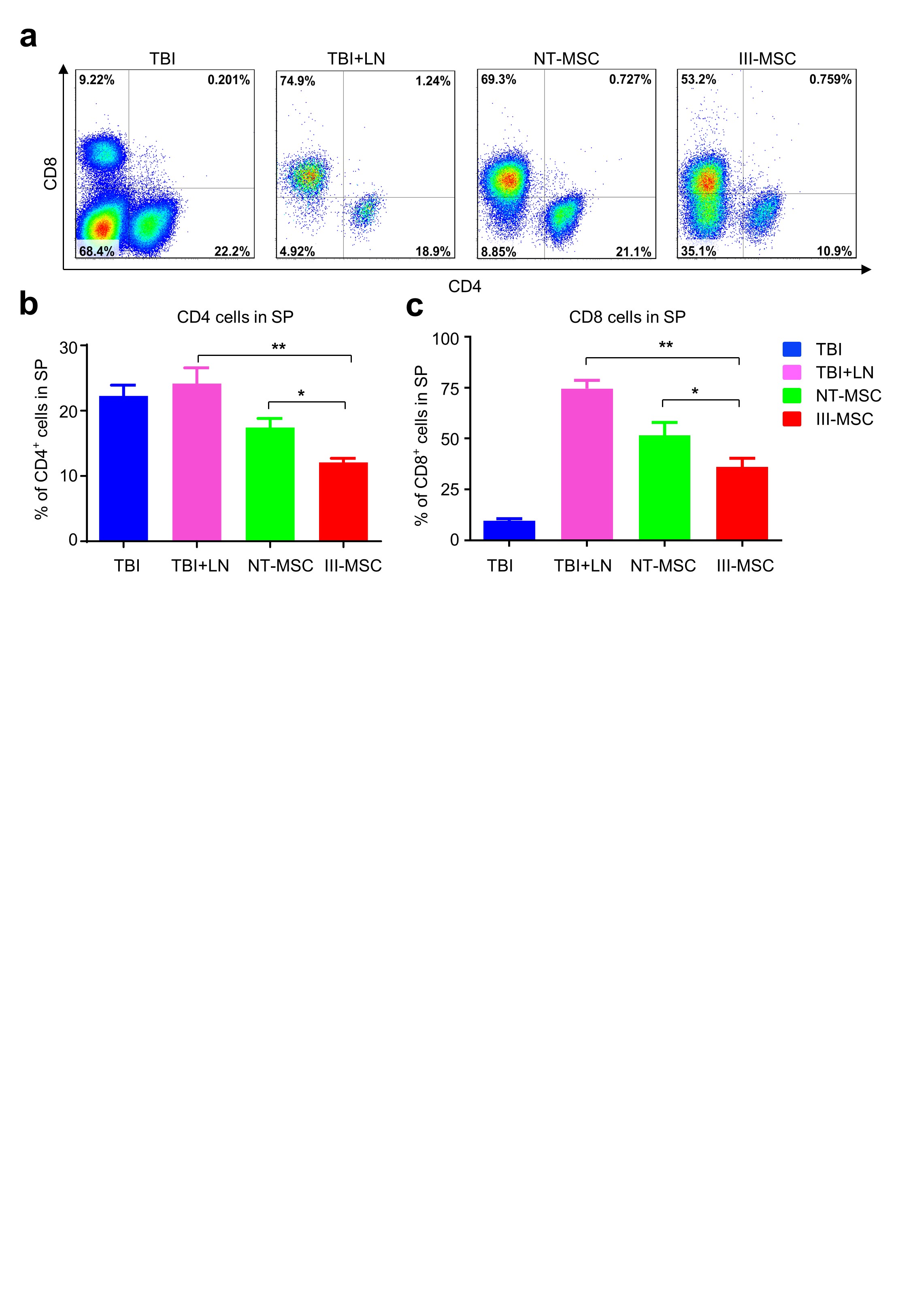

Supplement: Supplementary file 5 — Figure S4 [file CPR-53-e12862-s005.jpg]
